# Expectations of Social Consequences Impact Anticipated Involvement in Health-Risk Behavior During Adolescence

**DOI:** 10.1111/jora.12576

**Published:** 2020-09-10

**Authors:** Jack L. Andrews, John C. Flournoy, Garrett Ross, Kathryn L. Mills, Jessica E. Flannery, Arian Mobasser, Maureen Durnin, Shannon Peake, Philip A. Fisher, Jennifer H. Pfeifer

**Affiliations:** University College London; University of Oregon and Harvard University; University of Oregon and University of Florida; University of Oregon and University of Oslo; University of Oregon; University of Oregon; University of Oregon; University of Oregon; University of Oregon; University of Oregon

## Abstract

This study examined how individual differences in expectations of social consequences relate to individuals’ expected involvement in health-risk behaviors (HRBs). A total of 122 adolescents (aged 11–17) reported their expected involvement in a number of risk behaviors and whether or not they expect to be liked more or less by engaging in the behavior: the expected social benefit. Higher perceived social benefit was associated with higher anticipated involvement in said behavior. This relationship was stronger for adolescents who reported a higher degree of peer victimization, supporting the hypothesis that experiencing victimization increases the social value of peer interactions. Findings suggest that adolescents incorporate expectations of social consequences when making decisions regarding their involvement in HRBs.

## INTRODUCTION

Health-risk behaviors (HRBs), such as binge drinking and illicit substance use, cluster during the adolescent years (DuRant, Smith, Kreiter, & Krowchuk, 1999; van Nieuwenhuijzen et al., 2009). This increase in HRBs has fueled the stereotype that adolescents generally take more risks than children or adults. However, recent studies have suggested that adolescence is a time of risk sensitivity (van Duijvenvoorde et al., 2015), rather than a time of universal increases in risk-taking behavior. Risk sensitivity is an individual difference characterized as the degree to which individuals are risk seeking or risk averse. In particular, it has been suggested that risk-taking behaviors during adolescence are related to heightened social sensitivity, especially from peers (Blakemore & Mills, 2014). This theory predicts that an adolescent’s perception of the social outcome of engagement in a particular risky behavior will in part determine their likelihood of engaging in that behavior. In this paper, we test this prediction by building a model of adolescent risk taking that accounts for the perceived social consequences of engaging in a variety of risky behaviors.

### Current Models of Adolescent Risk Taking

Developmental scientists have proposed several theories as to why risk-taking behaviors increase during this period of life (Ciranka & Bos, 2019). Several theories focus on the heightened degree of sensation seeking, reward value, and peer influence (Steinberg, 2008). Here, we begin by elaborating on a number of the most prevalent models of adolescent risk taking, before focusing in on the impact that the peer environment has on decisions to engage in risk behaviors during adolescence.

Firstly, reward sensitivity models, like the dual systems model, attribute increased risk taking during adolescence to divergent patterns of developing motivational and cognitive control systems (Steinberg, 2010). A number of neuroscientific findings in the last decade are, however, inconsistent with the dual systems model (Crone & Dahl, 2012; Pfeifer & Allen, 2012). For example, one study found that at an individual level there was wide variation in the presence of a developmental mismatch in the timing of development between subcortical regions (involved in motivational processes) and prefrontal regions (involved in cognitive control), with a number of individuals showing no evidence of a mismatch at all (Mills, Goddings, Clasen, Giedd, & Blakemore, 2014). Further, the extent to which this mismatch existed was unrelated to individuals retrospectively self-reported engagement in risky behavior during adolescence (Mills et al., 2014). In contrast, in recent years, the imbalance model (Casey, Jones, & Hare, 2008) has explicitly moved away from the dichotomy inherent in the dual systems model and argued for a more nuanced understanding which accounts for variation in self-control across both content (e.g., emotions or actions) and context (e.g., in the presence of peers or parents; Casey, Galván, & Somerville, 2016).

Secondly, a more recent model of adolescent risk taking, the Lifespan Wisdom Model, challenges the notion of a universal imbalance between the development of brain systems underpinning motivation and cognitive control and delineates adaptive and maladaptive risk taking during adolescence (Romer, Reyna, & Satterthwaite, 2017). This model argues that most risk taking during adolescence is adaptive, allowing the individual to gain experience through exploration of the environment, particularly during ambiguous risk contexts (which is characterized by sensation seeking). In general, however, when risks are known, risk taking declines monotonically from childhood to adulthood (Tymula et al., 2012). The Lifespan Wisdom Model also argues for the incorporation of broader risk contexts such as “social conflicts,” with parents or peers, to be included in models of risk taking (Romer et al., 2017).

In turn, models which focus on the role of sensation seeking in accounting for this rise in risk-taking behavior between childhood and adulthood have also been widely discussed (Romer et al., 2017; Steinberg, 2008). One large cross-cultural study has shown that across a number of countries self-reported sensation seeking peaks during adolescence, at 19 years of age, before declining thereafter (Steinberg et al., 2018) and previous work has documented a positive association between sensation seeking, substance use, and sexual risk taking (Byck, Swann, Schalet, Bolland, & Mustanski, 2015; Quinn & Harden, 2013; Zhang, Zhang, & Shang, 2016). Work investigating the relationship between sensation seeking and peer influence has also shown, in a large sample of adolescents, that peer pressure to engage in marijuana and cigarette use had a greater effect on high sensation seekers (Slater, 2003). Subsequent research has also found that the frequency with which individuals (college students) associated with alcohol-using peers influenced the relationship between sensation seeking and alcohol consumption (Yanovitzky, 2006). Taken together, this suggests that social context has the potential to moderate the relationship between sensation seeking and engagement in HRBs.

In situations where risk-taking behaviors (e.g., HRBs) are perceived to have a high social value, individuals may be more likely to engage in these behaviors in order to reach their social goals (Blakemore & Mills, 2014; Crone & Dahl, 2012). This social motivation account of adolescent risk behavior is consistent with a value-based choice explanation of adolescent decision-making (Pfeifer & Berkman, 2018), which proposes that a single system integrates diverse value-laden inputs (which could include expectations of the social consequences/social motives) to inform choices. In line with this value-based approach, Do, Sharp, and Telzer (2019) apply an expected value-of-control model to adolescent risk taking, which argues that adolescent risk taking can be seen as adaptive and can require cognitive control, which is counter to existing dual systems models. For example, in situations where risk behaviors are not habitual (e.g., when engaging in a risk behavior for the first time), it is likely that individuals employ a degree of cognitive control to counter the habitual response (to avoid the risk behavior), particularly in contexts where risk engagement may be valued through social approval (Do et al., 2019).

In the present study, we are interested in understanding how expectations of social consequences, expecting to be liked or disliked, impact decisions to engage in HRBs during adolescence. Here, we build upon predictions made by social motivation and value-based explanations of adolescent decision-making. These accounts make the prediction that individuals who place a greater value on social approval may be more likely to engage in certain HRBs if they expect this to lead to increased social status and likability.

### Risk Taking and the Peer Environment

During adolescence, individuals undergo a period of substantial social reorientation where they begin to spend more time with their peers (Lam, McHale, & Crouter, 2014; Nelson, Jarcho, & Guyer, 2016) and establish their position within a widening and, often unstable, social network of friends (Hartl, Laursen, & Cillessen, 2015). Importantly, adaptively navigating one’s social environment during adolescence, in order to attain social status and good quality of friendships, has been shown to have particular benefits for one’s future social, psychological, and physical health (Almquist, 2009; van Harmelen et al., 2017). One mechanism via which individuals may attain a beneficial position within a social group is through normative social influence, which is conforming to others in order to be accepted or liked.

Previous work that is consistent with this view has shown that belonging to a peer group that engages in smoking or drinking increases an individual’s likelihood of smoking by 5.4 times and drinking by 1.9 times (Loke & Mak, 2013). Adolescents are also more likely to engage in HRBs such as excessive alcohol consumption, experimentation with illicit substances, and smoking when with their peers, compared to when alone (Reniers et al., 2017). Data from across three US government surveys have also shown that the risk of car accidents is greater for young drivers when they have a passenger in the car (Chen, Baker, Braver, & Li, 2000).

Adolescence might be a period of particular susceptibility to peer influence due to sensitivity to the negative effects of social rejection. For example, following experimentally induced social exclusion, 11- to 15-year-olds report a greater decrease in mood compared to adults (22–47; Sebastian, Viding, Williams, & Blakemore, 2010). Further, in a sample of 10- to 13-year-olds, individuals with low resistance to peer influence were more likely to take risks on a simulated driving task, following social exclusion, compared to individuals with high resistance to peer influence (Peake, Dishion, Stormshak, Moore, & Pfeifer, 2013). Social rejection sensitivity has also been studied in relation to an incentivized risk-taking task, where individuals knock on a virtual door to earn points (McCormick, Perino, & Telzer, 2018). With each knock, the facial expression of the resident turns from happy to angry; however, if the face becomes too angry, the door slams and all points are lost. Moderate social sensitivity on this task was related to adaptive decision-making (McCormick et al., 2018).

Prior work has also revealed an association between engagement in substance use and victimization during adolescence. For example, in a sample of 12- to 17-year-olds from South Africa, there was a significant association between substance use (e.g., tobacco, alcohol, and marijuana) and prior victimization experiences (Morojele & Brook, 2006). Further, in a large study of over four hundred thousand adolescents from California, USA, aged between 12 and 17 found that individuals who experienced high rates of victimization were twice as likely to frequently use substances such as tobacco, alcohol, and marijuana (Gilreath, Astor, Estrada, Benbenishty, & Unger, 2014). The authors suggest that these results should be considered within the context of adolescence as a period crucial for developing peer relationships.

Taken together, these findings suggest that engaging in HRBs is influenced by the perceived social consequences, which could include social risks. Social risks can be defined as any decision or action that might lead to peer exclusion, lowering one’s place in one’s social hierarchy, embarrassment, or loss of face (Allen & Badcock, 2003; Blakemore, 2018). We argue that when making decisions, the expectations of the social consequences associated with each decision need to be better incorporated into models of risk-taking behavior and that individual variation in the degree to which these expectations relate to expected positive social outcomes is a likely predictor of an individual’s involvement in HRBs. This theory suggests that individuals who engage in HRBs, in order to avoid social risks, are making decisions that minimize their overall risk when both are considered.

### Current Study

Previous work has shown that positive outcome expectancies (e.g., pleasure, winning money, feeling good about oneself) are positively associated with drug use while both positive and negative outcome expectancies are associated with heavy drinking (Katz, Fromme, & D’Amico, 2000). Yet, the literature surrounding how outcome expectancies and motivations drive engagement in risky sex is mixed. For example, in one such study risky sex was not predicted by outcome expectancies in a sample of young adults (mean age 18) but rather by past experience (Katz et al., 2000). However, individual differences in appetitive self-focused motivations predicted engagement in risky sex in a sample of young adults (Cooper, Shapiro, & Powers, 1998). In the present study, we examine how individual differences in expectations of the social consequences relate to an individual’s expectation of future involvement in risky behavior, during adolescence. We investigated the extent to which an individual’s perceptions of the social benefit—that is, the extent to which they expect to be liked less or more by others as a result of their behavioral choices, are associated with expected engagement in a number of HRBs including aggressive and illegal behavior; substance use; risky sex; and risky drinking. Our study is therefore capable of contributing to previous work by exploring whether or not engagement in risky sex behaviors are socially motivated in ways similar or dissimilar to other HRBs.

We hypothesize that individuals will incorporate their expectations of the social consequences associated with engagement in each HRB into their expected involvement in each HRB, such that expected involvement will be related to the perceived social benefit of engagement (hypothesis 1). We predict that individuals who believe they will be liked more by engaging in a HRB will be more likely to expect to perform that behavior, while individuals who believe that they will be liked less by engaging in said behavior will be less likely to do so. We subsequently explore whether this hypothesis varies depending on the type of health risk, across four risk domains: aggressive and illegal behavior; substance use; risky sex; and risky drinking. Additionally, we anticipate that this relationship between perceived social benefit and engagement will be moderated by a number of characteristics. We predict that individuals who report greater resistance to peer influence will show a diminished relationship between the perceived social benefit and engagement in risky behavior (hypothesis 2), which would be consistent with previous findings (Peake et al., 2013). We further predict that individuals who report greater fear of negative evaluation (hypothesis 3) and higher levels of peer victimization (hypothesis 4) will show a stronger relationship between perceived social benefit and engagement in HRBs. Evidence in support of the social augmentation hypothesis (Dishion, Piehler, & Myers, 2008) suggests that victimization experiences augment, or increase, the value of peer interactions—leading us to hypothesize that individuals with a history of peer victimization will be more sensitive to the social consequences associated with their involvement in HRBs. We further hypothesized that individuals with lower levels of self-esteem (hypothesis 5) will show a stronger relationship between perceived social benefit and engagement in HRBs. Previous evidence linking self-esteem to engagement in risk behaviors is varied. For example, one study found no relationship between self-confidence and risk taking (Bayat, Akbarisomar, Tori, & Salehiniya, 2019, while others show high self-esteem is related to increased risk behavior in a nonhealth context (Tian, Yuan, & Li, 2017) yet when related to HRBs, low self-esteem has been shown to contribute (Geckil & Dündar, 2011). Finally, we hypothesize that perceived social benefit will moderate the relationship between sensation seeking and engagement in risky behaviors, such that individuals who perceive the social benefit of risk taking to be high will show a stronger relationship between sensation seeking and engagement in HRBs (hypothesis 6).

## METHODS

### Participants

One hundred and eighty-three participants between the ages of 11 and 19 years were recruited as part of the *Teen Decisions Study* (TDS; Research Component 1 of P50 DA035763) from the Eugene/Springfield, Oregon (USA), metropolitan area. The sample included in this project was drawn from three separate populations recruited for the TDS study. Of the 183 participants, 76 were recruited through contact with the Eugene Department of Human Services (DHS) (TDS 1), 97 were recruited in the local community (TDS 2), and 10 were recruited through their involvement with juvenile justice (TDS 3). The DHS-based sample participants were recruited because of their involvement with the child welfare system (e.g., in foster care) and were contacted through a DHS liaison. The community-based sample participants were recruited through distributed flyers, outreach events organized by the laboratory, online advertisement, tabling, and word of mouth. The juvenile justice sample participants were recruited by recontacting participants who had previously participated in another study conducted at the University of Oregon called the SHARP study (Research Component 2 of P50 DA035763). Caregivers of participants from TDS 2 and TDS 3, and caseworkers of participants from TDS 1, provided informed consent, and all adolescent participants provided informed assent, in accordance with the Oregon Institutional Review Board and the DHS review board.

For the purposes of this analysis and to maintain sample homogeneity, we removed the small number of participants (N-10) who were recruited through the juvenile justice system. They were recruited during a period of time when we were experiencing difficulties recruiting through DHS, but are excluded here given that their experiences in the child welfare system were conceptually unique from other adolescents with a history of child welfare involvement (*N* = 76). For completeness, we report in the Supplementary Material the results of the same analyses conducted below when these 10 participants are included in the analyses. The results of these supplementary analyses are broadly similar to the main results presented here, despite only minor differences, primarily in the results of hypotheses 3 and 5. We only included participants who had complete data for each variable of interest. This resulted in a total of 122 participants, between the ages of 11 and 17, of which 50 participants were from the DHS sample (TDS 1) and 72 were from the community sample (TDS 2). Across both samples, the mean age of participants was 14 and there was an equal split of gender (61 females, 61 males). See Table 1 for participant demographics split by sample.

### Study Design

Data for this analysis were collected at two sessions, with the second session occurring roughly 3 weeks after the first session. This study formed part of a larger project, which also involved an MRI scan at the second session. Therefore, in order to prevent participant fatigue given the number of assessments, data collection was spread across an initial and second session. The session in which each questionnaire was administered differed slightly between the DHS and community sample. This is outlined in Table S1. We report Cronbach’s alpha scores, which assumes unidimensionality of constructs, for each measure and McDonald’s Omega hierarchical (ωh), which does not assume unidimensionality, where possible.

### Materials

#### Cognitive appraisal of risky events (CAR-E).

We used items from the expected involvement (EI) subscale of the Cognitive Appraisal of Risky Events (Fromme, Katz, & Rivet, 1997) to assess expected involvement in the following domains of risky behavior; aggressive and illegal behaviors, substance use, risky sex, and risky drinking. All items were rated on a 7-point scale: 1 = not at all likely to 7 = extremely likely to engage in the specific health-risking behavior over the next 6 months. Scores were computed for each risk domain by averaging responses from items corresponding to each of the four risk domains of interest within the CARE (see supplemental material for the risk items included): aggressive and illegal behavior (alpha 0.62; McDonald’s ωh 0.57), substance use (alpha 0.55; McDonald’s ωh 0.52), risky sex (alpha 0.72; McDonald’s ωh 0.65), and risky drinking (alpha 0.73).

#### Social appraisal of risky events (SARE).

We created an addendum to the CARE in which for each item tapped in the CARE participants indicated how much people would like them if they (a) engaged in the behavior (Social Benefit-Do) and if they (b) did not engage in the behavior (Social Benefit-Not). Items were rated on a 5-point scale: 1 = a lot less, 2 = less, 3 = no difference, 4 = more, and 5 = a lot more. Items posed in the negative (i.e., how much did people like them if they did not engage in the behavior) were reverse-scored, so that higher scores indicate higher perceived social benefit associated with engaging in risky behavior. We computed an average score for each scale (Social Benefit-Do/Not) by averaging the responses from items corresponding to each of the four risk domains of interest within the CARE—Social Benefit-Do scales: aggressive and illegal behavior (alpha 0.82; McDonald’s ωh 0.8), substance use (alpha 0.78), risky sex (alpha 0.75), and risky drinking (alpha 0.78); Social Benefit-Not scales: aggressive and illegal behavior (alpha 0.93; McDonald’s ωh 0.92), substance use (alpha 0.82), risky sex (alpha 0.84), and risky drinking (alpha 0.87).

#### Resistance to peer influence (RPI).

Resistance to Peer Influence (RPI). We scored each item on the 10-item RPI (Steinberg & Monahan, 2007) from 1 to 4 (reading left to right on the instrument) and reverse-scored items 2, 6, and 10. The mean score was calculated by summing the scores across the valid items and dividing them by the number of valid items (alpha, 0.50; McDonald’s ωh 0.54).

#### Fear of negative evaluation (BFNE-S).

The Brief Fear of Negative Evaluation (BFNE) is a 12-item questionnaire used to assess fear of negative evaluation. Each item is rated on a 5-point scale, ranging from 0 (not at all characteristic of me) to 4 (extremely characteristic of me). We calculated a mean score across the eight straightforwardly worded items because they have been reported to be “more reliable and valid indicators” (Weeks et al., 2005). This 8-item version is called the BFNE-5 (Carleton, McCreary, Norton, & Asmundson, 2006; alpha, 0.94; McDonald’s ωh 0.88).

#### Peer experiences questionnaire (RPEQ).

The Revised Peer Experiences Questionnaire (RPEQ) consists of 36 items that are scored on a 5-point scale: 1 = never, 2 = once or twice, 3 = a few times, 4 = about once a week, and 5 = a few times a week to assess bullying behavior and experiences of victimization (Prinstein, Boergers, & Vernberg, 2001). For the current project, we only used items that were worded such that the participant reports on being the victim, in order to get an indication of the participant’s perceived sense of being victimized by peers. Average scores were computed from the items regarding individuals’ overt and relational victimization experiences and then combined in order to create a total victimization score (alpha 0.86; McDonald’s ωh 0.64).

#### Need Threat Scale (NTS).

Participants played Cyber ball (Williams, Cheung, & Choi, 2000), a virtual ball tossing game, in which they are excluded from the game by two other players. Participants were led to believe they were playing against two other players; however, these players were programmed by the experimenter to exclude the participant. Participants were then asked the Need Threat Scale (NTS) and asked to indicate how they felt during the Cyber ball game. The NTS includes 12 items that are scored on a 5-point scale: 1 = not at all, 3 = moderately, 5 = very much so. Only the Self-esteem subscale is included in the current analysis. The items included in the Self-esteem subscale are reverse-scored, so that higher scores correspond with experiencing greater threat to an individual’s need for self-esteem (alpha 0.60; McDonald’s ωh 0.01).

#### Sensation seeking (BSSS).

The Brief Sensation Seeking Scale (BSSS) was adapted by Hoyle and colleagues from the Sensation Seeking Scale and tailored for adolescents (Hoyle, Stephenson, Palm-green, Lorch, & Donohew, 2002). It consists of eight pairs of statements, with one of the two statements associated with sensation-seeking behavior, and participants are asked to select the statement that best describes their preferences from of each pair. Sensation-seeking scores are calculated by summing together the total number of sensation-seeking statement selected from the eight pairs (alpha 0.68; McDonald’s ωh 0.88).

### Statistical Analyses

We employed a linear mixed-effects modeling approach to our data with the lme4 package in R (R Core Team, 2014). We take a model comparison approach whereby the model with the lowest Akaike information criterion (AIC) value that was significantly different (*p* < .05), as determined by a likelihood ratio test (LRT), from the less complex model was chosen. *p* values for model comparisons therefore represent likelihood ratio testing. Where we are interested in the interaction between two or more variables, we use LRTs to compare the interaction model against a simpler model where the interaction term of interest is entered as a fixed effect (*p* < .05). Where omnibus tests and model parameters are reported, *p* values for *F* and *t* statistics are approximated with the Satterthwaite method, which approximates the degrees of freedom, given that the null distributions of parameter estimates and test statistics are unknown (Kuznetsova, Brockhoff, & Christensen, 2017). All models were performed on the averaged scores derived from the subscales of each measure as detailed above, not at the item level. All models included age, gender, IQ, and group (community: TDS 2; child welfare services: TDS 1) as fixed factors. Please see the supplementary material (Table S2) for a comparison of models including and excluding age, gender, IQ, and group.

The first aim was to examine our hypothesis that there would be a relationship between expected involvement in risky behavior and the perceived social benefit of engaging in, or not engaging in, these risky behaviors. Expected involvement was measured with the CARE and the perceived social benefit of engaging in (Social Benefit-Do), or not engaging in (Social Benefit -Not) each of these behaviors was measured by the two questions asked in the SARE. In step one, we find the best fitting model that predicts expected involvement using the SARE (see Table 2 for models). In step two, we were interested to see whether an interaction between social benefit and risk domain, that is the type of risk behavior (aggressive and illegal, substance use, risky sex, or risky drinking), improved the model. We therefore compare our best fitting model from step one with a model incorporating the interaction with risk domain in step two.

We tested hypotheses 2–5 (see Table 2 for models) that this relationship would be moderated by a number of individual characteristics, such as resistance to peer influence, fear of negative evaluation, peer victimization, and fear of negative evaluation. We did this by comparing our best fitting model arising from the results of our first hypothesis, to a model that incorporated the individual difference of interest.

Our last hypothesis (6) investigated the relationship between sensation seeking and expected involvement in risky behaviors including the moderating effect of perceived social benefit on this relationship. We compared a model predicting expected involvement in risky behavior with sensation seeking against our null model. We further compared a model incorporating an interaction between sensation seeking, social benefit, and risk domain with our simpler model.

## RESULTS

### Hypothesis 1: Social Benefit of Risk Taking

In step one, model 3, which included age, gender, IQ, group, and Social benefit-do, best fitted the data. Across all models of interest, model 3 had the best model fit based on our AIC criteria. Model 3 provided a better fit to all simpler models; the null (X^2^(5) = 40.48, *p* = <.001) and a model (model 2) including just age, gender, and IQ (X^2^(1) = 29.85, *p* = <.001). Model 3 provided a better fit (lower AIC) to more complex models (model 5 and model 6) in which social benefit-do and social benefit-not were entered together either as main effects or as an interaction with each other, respectively. There was a significant correlation between the two subscales of the SARE: Social benefit-do and Social benefit-not (*r* = .51, *p* < .001).

In step two, we added risk domain (substance use, aggressive and illegal behavior, risky drinking, and risky sex) as an interaction term to our model. Model 7, which included age, gender, IQ, group, and an interaction between social benefit and risk domain, explained more variance in expected involvement than model 3 (X^2^(6) = 26.73, *p* = <.001).

We tested the significance of this interaction by comparing model 7 to a model where risk domain was entered, but not as an interaction (model 8). Model 7 outperformed model 8 (X^2^(3) = 19.41, *p* = <.001), revealing the additional benefit of the interaction between perceived social benefit and risk domain in explaining expected involvement. Therefore, our best fitting model (model 7) included age, gender, IQ, group, and an interaction between social benefit and risk domain.

**FIGURE 1:**
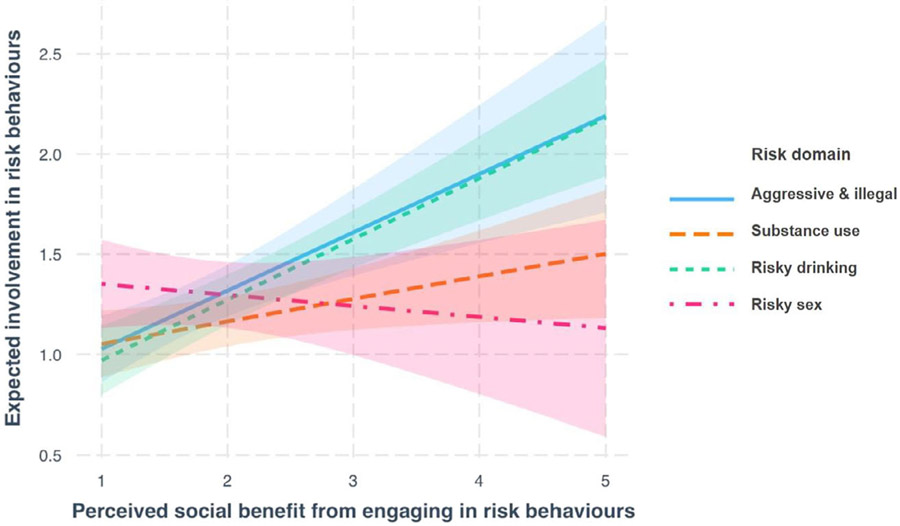
Relationship between perceived social benefit and expected involvement in risk behavior, broken down by risk domain.

Estimates for model 7 are found in Table 3. Omnibus tests on model 7 revealed a main effect of age, *F*(1,123.06) = 6.15, *p* = .014, social benefit, *F* (1, 380.46) = 19.62, *p* = <.001, risk domain, *F* (3, 350.60) = 5.21, *p* = .001, and a interaction of social benefit and risk domain, *F* (3, 356.92) = 6.67, *p* = <.001. To explore the interaction between perceived social benefit and risk domain, we plotted the relationship for each risk domain (Figure 1) and used simple slope analyses. The perceived social benefit from engaging in aggressive and illegal behaviors (*β* = 0.29, *p* < .001), substance use (*β* = 0.11, *p* = .03), and risky drinking (*β* = 0.30, *p* < .001) predicted expected involvement in these respective risk behaviors; however, this was not the case for risky sex (*β* = −0.05, *p* = .52) (Table 3).

### Correlations Between Variables of Interest

**FIGURE 2:**
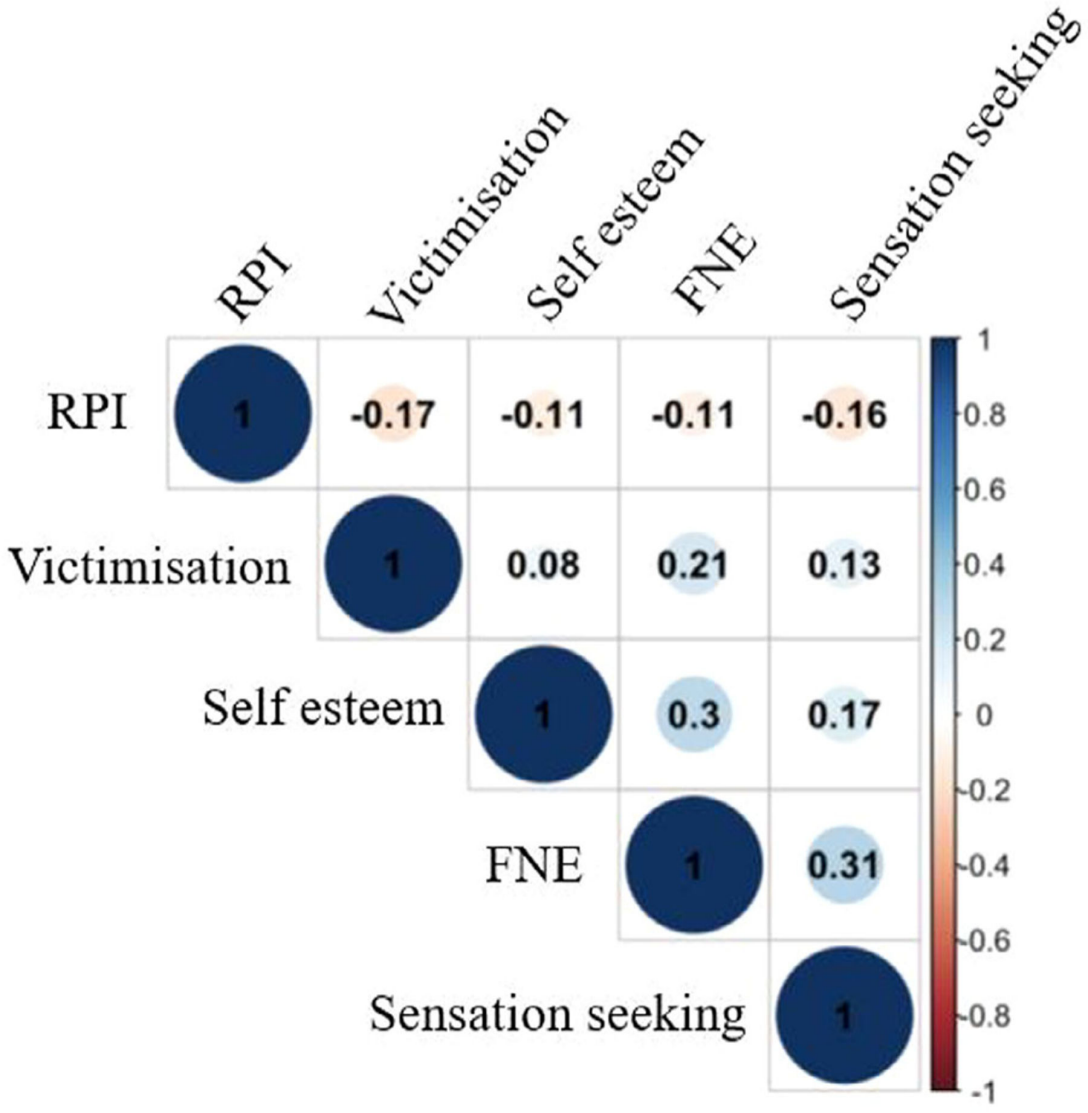
Correlation plot. Correlation plot between all subsequent variables of interest (FNE = fear of negative evaluation, RPI = resistance to peer influence). Overlaid numbers indicate the Pearson correlation coefficient.

We computed a correlation plot depicting the relationship between each subsequent variable of interest (see Figure 2).

### Hypothesis 2: Resistance to Peer Influence

A model including an interaction with resistance to peer influence (model 9) better fit the data compared to our simpler model (model 7; X^2^(8) = 21.90, *p* = .005). We further tested whether this model including the interaction with RPI explained more variance in expected involvement over a simpler model where RPI was entered, but not as an interaction (model 10). Model 9 provided a better model fit than model 10, revealing the additional benefit of the interaction between perceived social benefit, risk domain, and resistance to peer influence in explaining expected involvement. Therefore, model 9 which included the main effect of age, gender, IQ, and group, as well as an interaction between social benefit, risk domain, and resistance to peer influence best explains the data. Omnibus tests on model 9 revealed a main effect of age, *F*(1,122.15) = 11.17, *p* = .001, and social benefit, *F*(1,364.33) = 5.92, *p* = .02. All other fixed effects did not meet significance (*p* > .05).

### Hypothesis 3: Fear of Negative Evaluation

The more parsimonious model (model 7), which included age, gender, IQ, group, and an interaction between social benefit and risk domain, provided a better fit than a model including an interaction with fear of negative evaluation (model 11). Therefore, fear of negative evaluation does not interact with social benefit and risk domain in association with expected involvement in risk behaviors.

### Hypothesis 4: Peer Victimization

**FIGURE 3:**
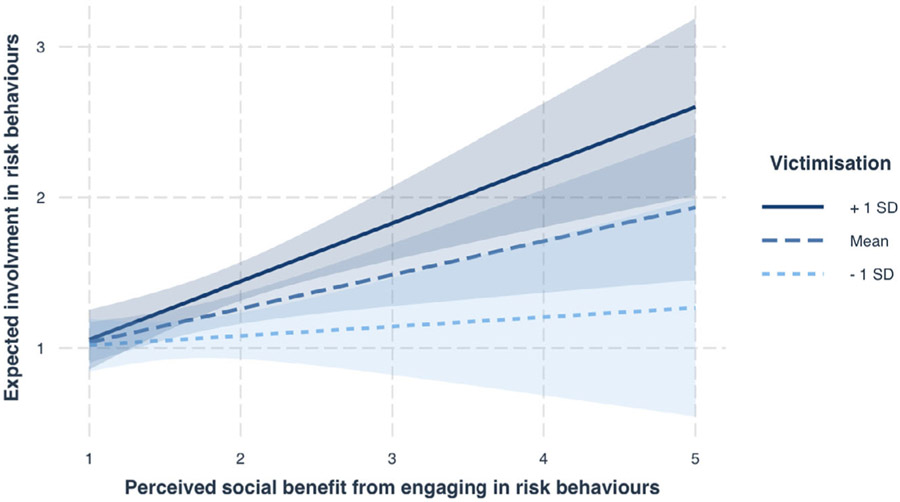
The moderating effect of victimization on the relationship between social benefit and expected involvement in risk behaviors.

A model including an interaction with victimization (model 12) outperformed our simpler model (model 7) (X^2^(8) = 26.43 *p* = <.001).We further tested whether this model including the interaction with victimization explained more variance in expected involvement over a simpler model where victimization was entered, but not as an interaction (model 13). Model 12 provided a better fit to our data than model 13 (X^2^(7) = 21.40 *p* = .003), revealing the additional benefit of the interaction between social benefit, risk domain, and victimization in explaining expected involvement. Omnibus tests on model 12 revealed main effects of age, *F* = 7.12, *p* = .008, and victimization, *F* =4.715, *p* = .031, and a significant interaction between social benefit and victimization, *F* (1,421.80) = 8.60, *p* = .004. All other fixed effects did not meet significance (*p* > .05). To explore the interaction between social benefit and victimization, we plotted the relationship (Figure 3) and used simple slope analyses. When victimization was high (+1 *SD*) individuals showed a strong relationship between social benefit and expected involvement (*β* = 0.39, *p* < .001), however, when victimization was low (−1 *SD*), the slope was not significant (*β* = 0.06, *p* = .55). Estimates for model 12 are shown in Table 4.

### Hypothesis 5: Self-Esteem

The more parsimonious model (model 7), which included age, gender, IQ, group, and an interaction between social benefit and risk domain, provided a better fit to a model including an interaction with self-esteem (model 14). Therefore, self-esteem does not interact with social benefit and risk domain in association with expected involvement in risk behaviors.

### Hypothesis 6: Sensation Seeking

**FIGURE 4:**
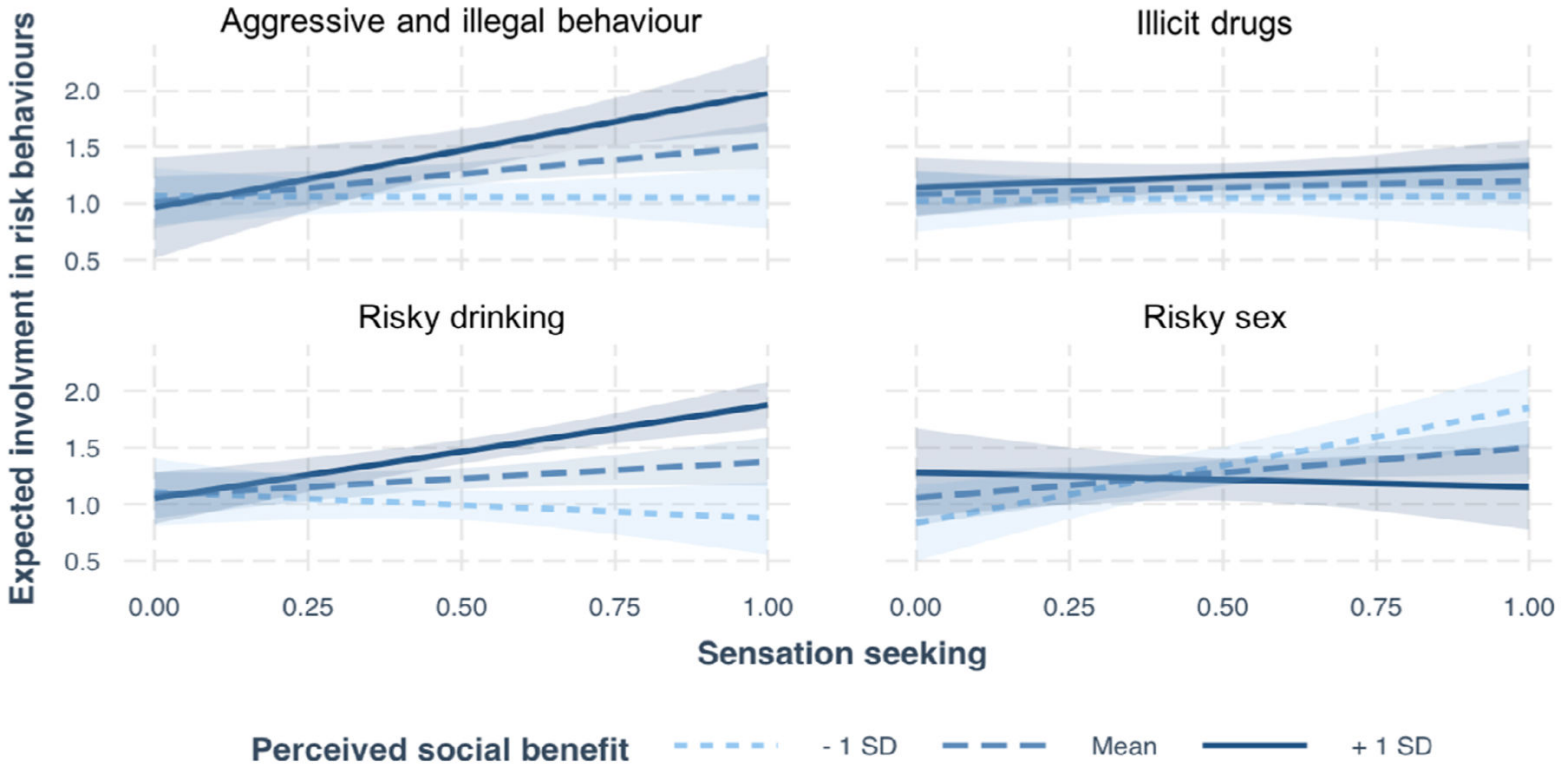
The moderating effect of social benefit on the relationship between sensation seeking and expected involvement in risk behaviors.

We built a model that predicted expected involvement in risky behaviors with sensation seeking (model 15). Model 15 provided a better fit than model 2 (which just included age, gender, IQ, and group; X^2^(2) = 12.94 *p* = <.001). We then added an interaction with perceived social benefit and risk domain to the model (model 16), which improved upon model 15 (X^2^(14) = 79.90, *p* = <.001). We tested the significance of including this three-way interaction by comparing model 16 to a simpler model (model 17). Model 16 outperformed model 17 (X^2^(7) = 26.19, *p* = <.001), revealing the additional benefit of the interaction between sensation seeking, perceived social benefit, and risk domain in explaining expected involvement. Therefore, our best fitting model (model 16) included the main effect of age, gender, IQ, and group, as well as an interaction between sensation seeking, perceived social benefit, and risk domain (see Table 5 for the estimates of model 16). Omnibus tests on model 16, revealed a main effect of age, *F*(1,118.85) = 4.51, *p* = .04, a two-way interaction of sensation seeking and risk domain, *F*(3, 359.0) = 5.55, *p* = <.001, and a three-way interaction of sensation seeking, social benefit, and risk domain, *F*(3,364.31) = 6.20, *p* = <.001 (Figure 4). Simple slope analyses revealed that when individuals perceived the social benefit of engaging in aggressive and illegal behaviors to be high (+1 *SD*) they show a strong relationship between sensation seeking and expected involvement (*b* = 1.01, *p* = .01); however, when they perceive the social benefit to be low, there is no significant relationship (*b* = −0.2, *p* = .94). This same pattern was observed for risky drinking. When individuals perceived the social benefit of engaging in risky drinking to be high (+1 *SD*), they show a strong relationship between sensation seeking and expected involvement (*β* = 0.82, *p* = <.001); however, when they perceive the social benefit to be low (−1 *SD*), there is no significant relationship (*β* = −0.23, *p* = .41). Simple slopes for drug use were not significant (*p* > .05). Finally, when the perceived social benefit of risky sex was low (−1 *SD*), there was a strong relationship between sensation seeking and expected involvement (*β* = 1.02, *p* = <.001); however, when they perceive the social benefit to be low (−1 *SD*), there is no significant relationship (*β* = −0.13, *p* = .71).

## DISCUSSION

In this study, we investigated the relationship between individual variation in expectations of social consequences and expected involvement in HRBs in a sample of adolescents. We show that the perceived social benefit of engagement explains variance in an individual’s expectations of engagement in aggressive and illegal behaviors, substance use, and risky drinking, but not risky sex, during adolescence. More specifically, we find that individuals who perceived the social benefit of engaging in these risky behaviors to be high (increased likability) were more likely to expect to engage in said behavior, while individuals who perceived the social benefit to be low (reduced likeability) were less likely to do so. This finding supports the theory that adolescents incorporate the social consequences when considering their likely engagement in a number of HRBs. These data support the view that individual difference in risk sensitivity, in particular sensitivity to social consequences, varies across adolescents—with some individuals placing greater weight on the social consequences than others. Across all models, we found that group membership (belonging to either the community or foster care sample) did not influence the findings. This suggests that the finding that the relationship between expected social benefit and anticipated involvement in HRBs in adolescence might apply across individuals with diverse backgrounds; however, this should be followed up in future studies using samples with other forms of diversity including greater racial and ethnic diversity.

Our findings build on previous work showing that knowledge of others’ decisions impacts decisions to take risks. In one study using a monetary risk-taking task (the Balloon Analogue Risk Task, in which individuals pump up a virtual balloon for money and with each pump the risk of it popping increases), individuals took more or less risky choices when presented with the knowledge that others had made high or low risky choices, respectively (Tomova & Pessoa, 2018). In another study which investigated the effects of social norms on simulated risky driving performance, adolescents (16–18 years) made more risky decisions when watched by risk-accepting versus risk-averse age-matched peers (Simons-Morton et al., 2014). These studies give weight to the social motivation model of risk taking, whereby individuals are motivated to conform to the social norms displayed by their peers. Our study adds to this literature by showing that it is not just adherence to a social norm that drives risky behavior during adolescence, but the associated expected social outcomes, specifically if you anticipate being liked more or less as a consequence.

During adolescence, one’s social and personal worth becomes increasingly dependent on peer relationships (O’Brien & Bierman, 1988). Therefore, engaging in behaviors that may lead to increased likeability may lead to positive psychological outcomes such as an increase in individuals perceived social and personal worth. We observe that adolescents’ expectations of engagement in HRBs are, in part, explained by how much they think others will like them for engaging (or dislike them for not engaging). Interestingly, we find this relationship to be true for all risk domains investigated except risky sexual behaviors. This finding is consistent with prior work showing that both positive and negative outcome expectancies did not predict engagement in risky sexual behavior (Katz et al., 2000). One possible explanation for this observation is that while sex is of course a social behavior, it is unlikely to occur in the presence of one’s peer group, in comparison with the other risky behaviors investigated, and therefore carries with it a different set of social evaluative concerns. In addition, societal expectations of what is “deviant” and what is not may be a contributing factor with regard to these findings. It is a possible that risky sex is perceived as less deviant than the other HRBs presented by the CARE, which might relate to our observed finding that social consequences do not relate to expected engagement in risky sex.

We subsequently found that individuals with a higher degree of peer victimization showed a stronger relationship between the perceived social benefit of, and expected involvement in, HRBs. In previous studies, chronic peer victimization and experimentally induced social exclusion have both been related to increased risk-taking behavior on laboratory tasks (Peake et al., 2013; Telzer, Miernicki, & Rudolph, 2018). There is also evidence that feeling nonprototypical, or dissimilar to one’s peers, augments adherence to group norms (Noel, Wann, & Branscombe, 1995), particularly in individuals who have a significant motivation for group acceptance (Steinel et al., 2010). Our data suggest that victimized individuals expect to make more socially risk-averse decisions, which may include placing greater weight on the social rather than the health consequences associated with a given decision, perhaps as a mechanism to attain likeability and peer acceptance. According to the social augmentation hypothesis (Dishion et al., 2008), victimization amplifies the role of deviant values in friendship formation. In support of this, there is evidence that individuals who have experienced peer rejection are likely to form friendships with other rejected peers, who support deviant behaviors (Dishion, Patterson, Stoolmiller, & Skinner, 1991). This theory suggests that chronic victimization augments the value of peer interaction. While we do not specifically ask about peers, the present findings support this idea that victimization increases social evaluative concern, leading to a greater weight being given to the social risk involved in a decision.

Although our findings suggest that the social consequences of HRBs play an important contribution to one’s expected involvement in a number of HRBs, one prevailing view is that HRBs during adolescence are in part explainable by a heightened degree of sensation seeking observed during this period of development. We show that the relationship between sensation seeking and expected involvement in HRBs is moderated by the perceived social benefit. With respect to aggressive and illegal behaviors, as well as risky drinking, individuals who perceive the social benefit of engaging in these behaviors (and/or social risks of not engaging) to be high, show an increased positive relationship between sensation seeking and engagement in the risky behavior. We find no such moderating effect for substance use, and an oppo-site effect for risky sexual behavior. When individuals believe they will be liked less by engaging in risky sex, their expected involvement in risky sex is better explained by sensation seeking than when they believe they will be liked more. Collectively, these findings demonstrate that engagement in risky behaviors is explained by an interaction between sensation seeking and concerns over the social consequences associated with expected involvement in HRBs.

### Limitations and Future Directions

Future work should build upon the findings reported here, to incorporate social expectations into measures of actual engagement in risk-taking behavior and experimental risk-taking tasks. The present study was unable to dissociate the effects of peer versus parent contributions to expectations of social consequences. In the present study, individuals were asked how much “people” would like them, rather than specifically peers or parents, therefore conflating these different sources of influence. There is a growing literature showing that, as well as peers, parents continue to be an important source of influence in adolescents decision processes (van Hoorn, McCormick, Rogers, Ivory, & Telzer, 2018; Qu, Fuligni, Galvan, & Telzer, 2015). Therefore, future work is needed in order to delineate the contribution of peers and parents in contributing to expectations of social consequences. In the present study, we took a model comparison approach, finding that a model including the perceived social benefit of engaging in HRBs outperformed similar models including the perceived social benefit of not engaging in HRBs. This may in part be due to shared variance, explaining anticipated involvement in HRBs, in our measures. That said, an interesting line of future work could be to further explore the qualitative differences between these measures.

In addition, the present study is unable to contribute to an understanding of how these social expectations impact expected involvement in HRBs across the lifespan. Therefore, in order to understand developmental trends, future work should consider assessing the impact of social expectations on risky decision-making across age. Additionally, our measure of the benefits associated with engagement in HRBs are strictly social in nature; therefore, we cannot infer beyond this to the contribution that more broader benefits, for example, financial gain, might have on engagement in these behaviors.

While the data presented here were self-report, these findings have significant implications for public health interventions, specifically supporting the suggestion that interventions aimed at reducing risky health behaviors such as binge drinking and substance misuse in young people should focus on changing social norms around risk behavior (Blakemore, 2018). However, interventions should be cautious of the possible iatrogenic effects of grouping deviant peers together (Gifford-Smith, Dodge, Dishion, & McCord, 2005). Rather, focusing on interventions that utilize a bottom-up, or peer-led, approach may have the most positive outcomes. This is evidenced by previous interventions that have utilized this approach reporting promising results for behaviors such as bullying (Paluck, Shepherd, & Aronow, 2016) and smoking (Campbell et al., 2008). More broadly, peers represent a potentially underused source of social change in current public health interventions; merely targeting the health consequences associated with a given risky decision ignores the broader social context that adolescents incorporate into their decisions regarding their engagement in (health) risk behaviors.

## CONCLUSION

The findings from the present study demonstrate the importance of incorporating perceptions of social consequences into models of HRB. Individual differences in the perception of the social consequences associated with a number of HRBs predicted adolescents expected involvement in these behaviors. This was true for aggressive and illegal behaviors, substance use, and risky drinking, but not risky sex. In addition, we find that this relationship is augmented in individuals with a history of victimization and that expectations of social consequences moderate the relationship between sensation seeking and expected involvement across a number of HRBs. This study provides an important contribution to the understanding of adolescent risk behavior.

Crucially, this study leads us to consider whether individuals, who are particularly sensitive to social consequences and also engage in HRBs, may in fact not be engaging in risky behavior *per se* but rather minimizing their overall risk exposure when aggregating across social and health risks. When incorporating an understanding of an individual’s expectations of the social consequences involved in these behaviors, individuals may therefore be acting to attain social acceptance, and avoid negative social outcomes.

## Supplementary Material

Supplementary Material Table S1 Depiction of the Timing of Each Assessment Tool Depending on the Sample Group
Table S2 Comparison of Models With and without Control Variables
Table S3 Participant Demographic Information
Table S4 Models Predicting Expected Involvement in Risky Behaviours
Table S5 Estimates for model 7
Table S6 Estimates for Model 16

## Figures and Tables

**TABLE 1:** Participant Demographic Information

Group	TDS 1	TDS 2
*N*	50	72
Age	Mean 14.0 (range 11.7–17.9)	Mean 14.1 (range 11.1–17.6)
Gender	Male = 29, Female = 21	Male = 32, Female = 40
IQ	Mean 98.8	Mean 108.0

**TABLE 2:** Models Predicting Expected Involvement in Risky Behaviors

Model name	Fixed effects	Random effects	AIC	R^2^ (marginal)
Hypothesis 1: Step one				
1 (null)	–	Subject ID	713.46	–
2	Age + Gender + IQ + Group	Subject ID	710.84	0.04
3	Age + Gender + IQ + Group + Social B-Do	Subject ID	682.98	0.11
4	Age + Gender + IQ + Group + Social B-Not	Subject ID	706.52	0.05
5	Age + Gender + IQ + Group + Social B-Do + Social B-Not	Subject ID	684.95	0.11
6	Age + Gender + IQ + Group + Social B-Do*Social B-Not	Subject ID	684.11	0.12
Hypothesis 1: Step two				
7	Age + Gender + IQ + Group + Social B-Do*Risk Domain	Subject ID	668.26	0.16
8	Age + Gender + IQ + Group + Social B-Do + Risk Domain	Subject ID	681.67	0.12
Hypothesis 2				
9	Age + Gender + IQ + Group + Social B-Do*Risk Domain*RPI	Subject ID	662.36	0.21
10	Age + Gender + IQ + Group + Social B-Do*Risk Domain + RPI	Subject ID	665.79	0.18
Hypothesis 3				
11	Age + Gender + IQ + Group + Social B-Do*Risk Domain*Negative Evaluation	Subject ID	668.30	0.19
Hypothesis 4				
12	Age + Gender + IQ + Group + Social B-Do*Risk Domain*Victimization	Subject ID	657.83	0.21
13	Age + Gender + IQ + Group + Social B-Do*Risk Domain + Victimization	Subject ID	665.22	0.17
Hypothesis 5				
14	Age + Gender + IQ + Group + Social B-Do*Risk Domain* Self-Esteem	Subject ID	671.10	0.18
Hypothesis 6				
15	Age + Gender + IQ + Group + Sensation seeking	Subject ID	699.90	0.08
16	Age + Gender + IQ + Group + Social B-Do*Risk Domain* Sensation Seeking	Subject ID	648.02	0.23
17	Age + Gender + IQ + Group + Social B-Do + Risk Domain + Sensation Seeking	Subject ID	660.21	0.12

Each model includes age, gender, IQ, and group (TDS 1: child welfare sample and TDS 2: community sample) as fixed effects. Social B-Do refers to the SARE scale measuring the perceived social benefit of engaging in the risk behavior. Social B-Not refers to the SARE scale measuring the perceived social benefit of not engaging in the risk behavior.

**TABLE 3:** Estimates for Model 7

*Fixed effects*	*Estimate*	SE	t	p
Intercept	0.32	0.4	0.80	.42
Age	0.05	0.02	2.48	.02
Gender	−0.08	0.06	−1.20	.23
IQ	−0.0	0.0	−0.92	.36
Group	−0.02	0.07	−0.31	.75
Social B-Do	0.29	0.07	3.98	<.001
Substance use	0.20	0.17	1.23	.22
Risky drinking	−0.07	0.17	−0.42	.68
Risky sex	0.67	0.21	3.20	<.001
Social B-Do*Substance use	−0.18	0.08	−2.11	.04
Social B-Do*Risky drinking	0.01	0.08	0.15	.89
Social B-Do*Risky sex	−0.35	0.11	−3.18	<.001
Total observations = 434				
*Random effects*	*Variance*			SD
Participant (intercept)	0.058			0.24
*Simple slopes*	*Estimate*	SE	t	p
Aggressive and illegal behaviors	0.29	0.07	4.02	<.001
Substance use	0.11	0.05	2.22	.03
Risky drinking	0.30	0.05	6.25	<.001
Risky sex	−0.05	0.08	−0.64	.52

This model included age, gender, IQ, group, and an interaction between social benefit and risk domain.

**TABLE 4:** Estimates for Model 12

*Fixed effects*	*Estimate*	SE	t	p
Intercept	0.92	0.56	1.63	.10
Age	0.05	0.02	2.67	<.01
Gender	−0.06	0.06	−1.04	.30
IQ	−0.00	0.00	−1.24	.22
Group	0.01	0.07	0.14	.89
Social B-Do	−0.33	0.25	−1.31	.19
Substance use	−0.41	0.55	−0.75	.46
Risky drinking	−0.16	0.56	−0.30	.78
Risky sex	1.53	0.83	1.85	.07
Victimization	−0.14	0.12	−1.17	.24
Social B-Do*Substance use	0.37	0.30	1.23	.21
Social B-Do*Risky drinking	0.23	0.30	0.78	.44
Social B-Do*Risky sex	−0.37	0.50	−0.80	.42
Social B-Do*Victimization	0.16	0.07	2.41	.02
Substance use*Victimization	0.16	0.15	1.01	.32
Risky drinking*Victimization	0.00	0.16	0.02	.99
Risky sex*Victimization	−0.30	0.25	−1.22	.22
Social B- Do*Substance use*Victimization	−0.14	0.08	−1.75	.08
Social B-Do*Risky drinking*Victimization	−0.05	0.08	−0.58	.56
Social B-Do*Risky sex*Victimization	0.04	0.14	0.30	.77
Total Observations = 434				
*Random effects*	*Variance*			SD
Participant (intercept)	0.05			0.22
*Simple slopes*	*Estimate*	SE	t	p
Victimization (−1 *SD*)	0.06	0.11	0.59	.55
Victimization (mean)	0.22	0.07	3.07	<.001
Victimization (+1 *SD*)	0.39	0.09	4.16	<.001

This model included age, gender, IQ, group, and an interaction between social benefit, risk domain, and victimization.

**TABLE 5:** Estimates for Model 16

*Fixed effects*	*Estimate*	SE	t	p
Intercept	0.80	0.47	1.71	.09
Age	0.04	0.02	2.10	.04
Gender	−0.10	0.06	−1.63	.11
IQ	−0.00	0.00	−0.65	.52
Group	−0.04	0.07	−0.60	.57
Sensation seeking	−0.71	0.50	−1.43	.15
Social benefit-do	−0.07	0.17	−0.40	.69
Substance use	−0.20	0.36	−0.55	.58
Risky drinking	−0.00	0.36	0.01	.99
Risky sex	−0.59	0.41	−1.43	.15
Sensation seeking*Social B-Do	0.62	0.28	2.24	.03
Sensation seeking*Substance use	0.67	0.66	1.01	.31
Sensation seeking*Risky drinking	−0.21	0.64	−0.33	.74
Sensation seeking*Risky sex	2.49	0.73	3.40	<.001
Social B-Do*Substance use	0.13	0.19	0.71	.48
Social B-Do*Risky drinking	0.03	0.19	0.18	.86
Social B-Do*Risky sex	0.33	0.23	1.46	.15
Sensation seeking* Social B-Do*Substance use	−0.54	0.33	−1.60	.11
Sensation seeking* Social B-Do*Risky drinking	0.01	0.32	0.02	.99
Sensation seeking* Social B-Do*Risky sex	−1.30	0.39	−3.34	<.001
Total Observations = 434				
*Random effects*	*Variance*			SD
Participant (intercept)	0.057			0.24
*Simple slopes*	*Estimate*	SE	t	p
Aggressive and illegal behaviors				
Social B-Do (−1 *SD*)	−0.2	0.24	−0.07	.94
Social B-Do (mean)	0.5	0.19	2.56	.01
Social B-Do (+1 *SD*)	1.01	0.34	2.82	.01
Substance use				
Social B-Do (−1 *SD*)	0.04	0.27	0.16	.87
Social B-Do (mean)	0.12	0.18	0.65	.52
Social B-Do (+1 *SD*)	0.19	0.22	0.88	.38
Risky drinking				
Social B-Do (−1 *SD*)	−0.23	0.28	−0.82	.41
Social B-Do (mean)	0.30	0.19	1.56	.12
Social B-Do (+1 *SD*)	0.82	0.19	4.30	<.001
Risky sex				
Social B-Do (−1 *SD*)	1.02	0.30	3.37	<.001
Social B-Do (mean)	0.44	0.21	2.08	.04
Social B-Do (+1 *SD*)	−0.13	0.34	−0.37	.71

This model included the main effect of age, gender, IQ and group, as well as an interaction between sensation seeking, perceived social benefit, and risk domain.
