# Supplementary material for: Expectations of Social Consequences Impact Anticipated Involvement in Health-Risk Behavior During Adolescence
Source: J Res Adolesc. Author manuscript; Available in PMC 2021 Dec 1. (PMC8494461; doi:10.1111/jora.12576)
Supplement: Supplementary Material — Table S1 Depiction of the Timing of Each Assessment Tool Depending on the Sample Group Table S2 Comparison of Models With and without Control Variables Table S3 Participant Demographic Information Table S4 Models Predicting Expected Involvement in Risky Behaviours Table S5 Estimates for model 7 Table S6 Estimates for Model 16 [file NIHMS1729813-supplement-Supplementary_Material.docx]

**Supplemental material**

|  |  | **TDS1** | | **TDS2** | | |  |
| --- | --- | --- | --- | --- | --- | --- | --- |
| **Measure** | **Acronym** | **Session 1** | **Session 2** | **Session 1** | **Session 2** | |  |
| Resistance to Peer Influence | RPI | x |  | x |  | |  |
| Cognitive Appraisal of Risky Events - Revised - Expected Involvement | CARE-EI | x |  |  | x | |  |
| Social Appraisal of Risky Events | SARE | x |  | x |  | |  |
| Peer Experiences Questionnaire - Revised | PEQ - R |  | x |  | x | |  |
| Brief Sensation Seeking Scale | BSSS |  | x |  | x | |  |
| Need Threat Scale | NTS |  | x |  | x | |  |
| Brief Fear of Negative Evaluation | BFNE |  | x |  | x | |  |
| Wechsler's Abbreviated Scale for Intelligence | WASI |  | x |  | x | |  |
| **Table S1:** Depiction of the Timing of Each Sssessment Tool Depending on the Sample Group | | | | | |  | |

**Risk behaviours**

Risk behaviours from the CARE included in analyses. For each of these behaviours individuals reported their expected involvement (CARE) and how much people would like them if they (a) engaged in the behaviour and (b) didn’t engage in the behaviour (SARE).

**Illicit drug use**

Trying drugs other than alcohol or marijuana

Smoking Marijuana

**Aggressive and illegal behaviours**

Driving after drinking alcohol

Damaging/destroying public property

Hitting someone with a weapon or object

Slapping someone

Punching or hitting someone with fist

**Risky sexual activities**

Sex without protection against pregnancy

Sex without protection against STD’s

Sex with someone I have just met or don’t know well

**Risky drinking**

Drinking more than 5 alcoholic beverages

Playing drinking games

|  | | |  |  |  |
| --- | --- | --- | --- | --- | --- |
| Model Name | Fixed Effects | Random Effects | AIC | AIC  of model without control variables | MLT |
| **Hypothesis 1: Step one** | | |  |  |  |
| 1 (null) |  | Subject ID | 713.46 | - | - |
| 2 | Age + Gender + IQ + Group | Subject ID | 710.84 | - | - |
| 3 | Age + Gender + IQ + Group + Social B-Do | Subject ID | 682.98 | 682.49 | >.05 |
| 4 | Age + Gender + IQ + Group + Social B-Not | Subject ID | 706.52 | 709.46 | <.05* |
| 5 | Age + Gender + IQ + Group + Social B-Do + Social B-Not | Subject ID | 684.95 | 684.32 | >.05 |
| 6 | Age + Gender + IQ + Group + Social B-Do*Social B-Not | Subject ID | 684.11 | 683.89 | >.05 |
| **Hypothesis 1: Step two** | | |  |  |  |
| 7 | Age + Gender + IQ + Group + Social B-Do*Risk Domain | Subject ID | 668.26 | 668.41 | >.05 |
| 8 | Age + Gender + IQ + Group + Social B-Do + Risk Domain | Subject ID | 681.67 | 681.41 | >.05 |
| **Hypothesis 2** | | |  | |  |
| 9 | Age + Gender + IQ + Group + Social B-Do*Risk Domain*RPI | Subject ID | 662.36 | 665,26 | <.05* |
| 10 | Age + Gender + IQ + Group + Social B-Do*Risk Domain + RPI | Subject ID | 662.79 | 664.65 | >.05 |
| **Hypothesis 3** | | |  | |  |
| 11 | Age + Gender + IQ + Group + Social B-Do*Risk Domain*Negative Evaluation | Subject ID | 668.30 | 669.40 | >.05 |
| **Hypothesis 4** | | |  | |  |
| 12 | Age + Gender + IQ + Group + Social B-Do*Risk Domain*Victimisation | Subject ID | 657.83 | 658.89 | >.05 |
| 13 | Age + Gender + IQ + Group + Social B-Do*Risk Domain + Victimisation | Subject ID | 665.22 | 666.60 | >.05 |
| **Hypothesis 5** | | |  | |  |
| 14 | Age + Gender + IQ + Group + Social B-Do*Risk Domain* Self Esteem | Subject ID | 671.10 | 672.05 | >.05 |
| **Hypothesis 6** | | |  | |  |
| 15 | Age + Gender + IQ + Group + Sensation seeking | Subject ID | 699.90 | 702.32 | <.05* |
| 16 | Age + Gender + IQ + Group + Social B-Do*Risk Domain* Sensation Seeking | Subject ID | 648.02 | 657.72 | >.05 |
| 17 | Age + Gender + IQ + Group + Social B-Do + Risk Domain + Sensation Seeking | Subject ID | 660.21 | 661.43 | >.05 |
| **Table S2: Comparison of Models With and without Control Variables.** Models including control variables (age, gender, IQ and group) are compared with models excluding these control variables. Social B-Do refers to the SARE scale measuring the perceived social benefit of engaging in the risk behaviour. Social B-Not refers to the SARE scale measuring the perceived social benefit of not engaging in the risk behaviour. MLT refers to maximum likelihood tests and the p values correspond to Chi Square values (* signifies significance where p<.05). | | | | | |

**Models on all data: including participants involved in the juvenile justice system**

The following analyses are conducted on the complete sample, including the additional 10 participants with involvement in the juvenile justice system (see table S3 for demographics). Each model and its associated AIC and marginal R^2^ values are reported in table S4.

|  | | |  |
| --- | --- | --- | --- |
| Group | **TDS 1** | **TDS 2** | **TDS 3** |
| *N* | 50 | 72 | 10 |
| Age | Mean 14.0 (Range 11.7 – 17.9) | Mean 14.1 (Range 11.1 – 17.6) | Mean 16.36 (Range 14.45 – 17.80) |
| Gender | Male=29, Female=21 | Male=32, Female=40 | Male = 10, Female =0 |
| IQ | Mean 98.8 | Mean 108.0 | Mean 106.2 |
| **Table S3:** **Participant Demographic Information** | | |  |

**Hypothesis 1: Social benefit of risk taking**

In step one, model 3, which included age, gender, IQ, group and Social benefit-do, best fitted the data. Across all models of interest, model 3 had the best model fit based on our AIC criteria. Model 3 provided a better fit to all simpler models; the null (X^2^(5)=47.26, *p*=<.001) and a model (model 2) including just age, gender and IQ (X^2^(1)=31.52, *p* =<.001).

In step two, we added risk domain (substance use, aggressive & illegal behaviour, risky drinking, and risky sex) as an interaction term to our model. Model 7,which included age, gender, IQ, group and an interaction between social benefit and risk domain, explained more variance in expected involvement than model 3 (X^2^(6)=28.98, *p* =<.001).

We tested the significance of this interaction by comparing model 7 to a model where Risk Domain was entered, but not as an interaction (model 8). Model 7 outperformed model 8 (X^2^(3)=19.77, *p* =<.001), revealing the additional benefit of the interaction between perceived social benefit and risk domain in explaining expected involvement. Therefore, our best fitting model (model 7), included age, gender, IQ, group and an interaction between social benefit and risk domain.

Estimates for model 7 are found in table S5. Omnibus tests on model 7, revealed a main effect of age (*F*(1, 134.02)=9.29, *p* =.003), social benefit (*F* (1, 372.43)=17.29, *p* =<.001), risk domain (*F* (3, 392.85)=5.21, *p* =0.002) and a interaction of social benefit and risk domain (*F* (3, 400.43)=6.75, *p* =<.001). To explore the interaction between perceived social benefit and risk domain we used simple slope analyses. The perceived social benefit from engaging in aggressive and illegal behaviours (*β* = 0.24, *p* <.001), substance use (*β* = 0.11, *p* =.05) and risky drinking (*β* = 0.35, *p* <.001) predicted expected involvement in these respective risk behaviours, however this was not the case for risky sex (*β* = -0.06, *p* =.54) (table 2).

**Correlations between variables of interest**

We computed a correlation plot depicting the relationship between each subsequent variable of interest (see Figure 2).

**Hypothesis 2: Resistance to peer influence**

A model including an interaction with resistance to peer influence (model 9) better fit the data compared to our simpler model (model 7) (X^2^(8)=19.99, *p* =0.01). We further tested whether this model including the interaction with RPI explained more variance in expected involvement over a simpler model where RPI was entered, but not as an interaction (model 10). Model 9 provided a better model fit than model 10, revealing the additional benefit of the interaction between perceived social benefit, risk domain, and resistance to peer influence in explaining expected involvement. Therefore, model 9 which included the main effect of age, gender, IQ and group, as well as an interaction between social benefit, risk domain, and resistance to peer influence best explains the data. Omnibus tests on model 9 revealed a main effect of age (*F* (1, 132.37)=15.70, *p* =.001) and social benefit (*F* (1, 368.55)=7.04, *p* =0.008) and a significant interaction between social benefit and RPI (*F* (1, 358.16)=4.37, *p* =0.03). We did not observe this significant interaction in our main analysis, in which the juvenile justice participants were excluded. All other fixed effects did not meet significance (*p* >.05).

**Hypothesis 3: Fear of negative evaluation**

The model including an interaction with fear of negative evaluation (model 11) provided a better fit than the simpler model (model 7), which included age, gender, IQ, group and an interaction between social benefit and risk domain (X^2^(8)=27.41, *p* =<0.001). Therefore, when in including the juvenile justice participants, we find that a model including an interaction with fear of negative evaluation better fits the data compared with our simpler model– this was not observed in our main analysis in which the juvenile justice participants were excluded. For completeness we then built an additional model (Model 11b) to compare model 11 with, in which fear of negative evaluation was included as a main effect. Model 11 (AIC: 838.26) outperformed our additional model (Model 11b; AIC: 842.28). Omnibus tests on model 11 revealed a main effect of age (*F* (1, 127.95)=6.62, *p* =0.01) and a significant interaction between social benefit, risk domain and fear of negative evaluation (*F* (3, 400.53)=7.04, *p* =0.01).

**Hypothesis 4: Peer victimisation**

A model including an interaction with victimisation (model 12) outperformed our simpler model (model 7) (X^2^(8)=21.55 *p* =0.005). We further tested whether this model including the interaction with victimisation explained more variance in expected involvement over a simpler model where victimisation was entered, but not as an interaction (model 13). Model 13 provided a better fit to our data than model 12 (X^2^(8)=15.99 *p* =0.04). Therefore, when the juvenile justice participants are included we find the best fitting model to be a model in which victimisation is included as a main effect not an interaction with social benefit. Omnibus tests on model 13 revealed main effects of age (*F* (1, 132.34)=11.06, *p* =.001), social benefit (*F* (1, 366.63)=15.13, *p* =<0.001), risk domain (*F* (3, 395.63)=4.99, *p* =.002), victimisation (*F* (1, 138.76)=5.68, *p* =.02), and a significant interaction between social benefit and risk domain (*F* (3, 402.27)=6.54 *p* =<0.001). All other fixed effects did not meet significance (*p* >.05).

**Hypothesis 5: Self esteem**

A model including an interaction with self-esteem (model 14) outperformed the simpler model (model 7), which included age, gender, IQ, group and an interaction between social benefit and risk domain (X^2^(8)=17.37 *p* =0.03). For completeness we then built an additional model (Model 14b) to compare model 14 with, in which self-esteem was included as a main effect. Model 14 outperformed our additional model (Model 14b; AIC: 849.28). Omnibus tests on model 14 revealed a main effect of age (*F* (1, 133.58)=9.7, *p* =0.002) and a significant interaction between social benefit and self-esteem (*F* (1, 329.35)=5.73, *p* =0.02). All other fixed effects did not meet significance (*p* >.05).

**Hypothesis 6: Sensation seeking**

We built a model that predicted expected involvement in risky behaviours with sensation seeking (model 15). Model 15 provided a better fit than model 2 (which just included age, gender, IQ and group) (X^2^(1)=12.72 *p* =<0.001). We then added an interaction with perceived social benefit and risk domain to the model (model 16), which improved upon model 15 (X^2^(14)=83.57, *p* =<0.001). We tested the significance of including this three-way interaction by comparing model 16 to a simpler model, in which sensation seeking was added as a main effect not an interaction term (model 17). Model 16 outperformed model 17 (X^2^(7)=26.66, *p* =<0.001), revealing the additional benefit of the interaction between sensation seeking, perceived social benefit, and risk domain in explaining expected involvement. Therefore, our best fitting model (model 16) included the main effect of age, gender, IQ and group, as well as an interaction between sensation seeking, perceived social benefit, and risk domain (see table S6 for the estimates of model 16). Omnibus tests on model 16, revealed a main effect of age (*F* (1, 130.22)=7.98, *p* =.04), a two way interaction of sensation seeking and risk domain (*F* (3, 404.38)=5.14, *p* =0.002) and a three way interaction of sensation seeking, social benefit, and risk domain (*F* (3, 410.61=5.86, *p* =<0.001). Simple slope analyses are reported in table S6.

| **Models predicting expected involvement in risky behaviours** | | |  |  |
| --- | --- | --- | --- | --- |
| Model Name | Fixed Effects | Random Effects | AIC | R^2^ (marginal) |
| **Hypothesis 1: Step one** | | |  |  |
| 1 (null) |  | Subject ID | 903.91 | - |
| 2 | Age + Gender + IQ + Group | Subject ID | 896.17 | 0.05 |
| 3 | Age + Gender + IQ + Group + Social B-Do | Subject ID | 866.65 | 0.11 |
| 4 | Age + Gender + IQ + Group + Social B-Not | Subject ID | 891.86 | 0.06 |
| 5 | Age + Gender + IQ + Group + Social B-Do + Social B-Not | Subject ID | 868.56 | 0.11 |
| 6 | Age + Gender + IQ + Group + Social B-Do*Social B-Not | Subject ID | 868.97 | 0.12 |
| **Hypothesis 1: Step two** | | |  |  |
| 7 | Age + Gender + IQ + Group + Social B-Do*Risk Domain | Subject ID | 849.67 | 0.16 |
| 8 | Age + Gender + IQ + Group + Social B-Do + Risk Domain | Subject ID | 863.44 | 0.13 |
| **Hypothesis 2** | | | |  |
| 9 | Age + Gender + IQ + Group + Social B-Do*Risk Domain*RPI | Subject ID | 845.68 | 0.20 |
| 10 | Age + Gender + IQ + Group + Social B-Do*Risk Domain + RPI | Subject ID | 842.59 | 0.18 |
| **Hypothesis 3** | | | |  |
| 11 | Age + Gender + IQ + Group + Social B-Do*Risk Domain*Negative Evaluation | Subject ID | 838.26 | 0.21 |
| 11b | Age + Gender + IQ + Group + Social B-Do*Risk Domain + Negative Evaluation | Subject ID | 842.28 | 0.18 |
| **Hypothesis 4** | | | |  |
| 12 | Age + Gender + IQ + Group + Social B-Do*Risk Domain*Victimisation | Subject ID | 844.13 | 0.20 |
| 13 | Age + Gender + IQ + Group + Social B-Do*Risk Domain + Victimisation | Subject ID | 844.11 | 0.20 |
| **Hypothesis 5** | | | |  |
| 14 | Age + Gender + IQ + Group + Social B-Do*Risk Domain* Self Esteem | Subject ID | 848.34 | 0.19 |
| 14b | Age + Gender + IQ + Group + Social B-Do*Risk Domain + Self Esteem | Subject ID | 849.28 | 0.17 |
| **Hypothesis 6** | | | |  |
| 15 | Age + Gender + IQ + Group + Sensation seeking | Subject ID | 885.45 | 0.17 |
| 16 | Age + Gender + IQ + Group + Social B-Do*Risk Domain* Sensation Seeking | Subject ID | 829.88 | 0.23 |
| 17 | Age + Gender + IQ + Group + Social B-Do + Risk Domain + Sensation Seeking | Subject ID | 842.54 | 0.18 |
| **Table S4: Models Predicting Expected Involvement in Risky Behaviours.** Each model includes age, gender, IQ and group (TDS 1: child welfare sample and TDS 2: community sample – TDS 3 are included in the same group at TDS 2 for these analyses given the extremely low sample size) as fixed effects. Social B-Do refers to the SARE scale measuring the perceived social benefit of engaging in the risk behaviour. Social B-Not refers to the SARE scale measuring the perceived social benefit of not engaging in the risk behaviour. | | | | |

| **Fixed effects** | **Estimate** | ***SE*** | ***t*** | ***p*** |
| --- | --- | --- | --- | --- |
| Intercept | 0.38 | 0.4 | 0.94 | .35 |
| Age | 0.06 | 0.02 | 3.05 | <.01 |
| Gender | -0.06 | 0.07 | -0.84 | .40 |
| IQ | -0.00 | 0.0 | -1.54 | .13 |
| Group | 0.00 | 0.07 | 0.01 | .99 |
| Social B-Do | 0.24 | 0.08 | 3.05 | <.01 |
| Substance use | 0.14 | 0.19 | 0.74 | .46 |
| Risky drinking | -0.19 | 0.19 | -1.07 | .68 |
| Risky sex | 0.65 | 0.23 | 2.83 | <.01 |
| Social B-Do*Substance use | -0.13 | 0.09 | -1.38 | .17 |
| Social B-Do*Risky drinking | 0.11 | 0.09 | 1.17 | .24 |
| Social B-Do*Risky sex | -0.30 | 0.12 | -2.51 | .01 |
| Total Observations = 474 | | | | |
| **Random effects** | **Variance** | | ***S.D.*** | |
| Participant (intercept) | 0.048 | | 0.22 | |
| **Simple slopes** | **Estimate** | ***SE*** | ***t*** | ***p*** |
| Aggressive and illegal behaviours | 0.24 | 0.08 | 3.05 | <.001 |
| Substance use | 0.11 | 0.06 | 2.00 | 0.05 |
| Risky drinking | 0.35 | 0.05 | 6.57 | <.001 |
| Risky sex | -0.06 | 0.09 | -0.61 | .54 |

**Table S5: Estimates for model 7.** This model included age, gender, IQ, group and an interaction between social benefit and risk domain.

| **Fixed effects** | **Estimate** | ***SE*** | ***t*** | ***p*** |
| --- | --- | --- | --- | --- |
| Intercept | 0.70 | 0.49 | 1.41 | .16 |
| Age | 0.05 | 0.02 | 2.82 | <.01 |
| Gender | -0.08 | 0.07 | -1.26 | .21 |
| IQ | -0.00 | 0.00 | -1.31 | .19 |
| Group | -0.02 | 0.07 | -0.20 | .86 |
| Sensation seeking | -0.44 | 0.55 | -0.80 | .43 |
| Social Benefit-Do | -0.01 | 0.19 | -0.06 | .95 |
| Substance use | -0.12 | 0.40 | -0.29 | .77 |
| Risky drinking | 0.31 | 0.40 | 0.78 | .43 |
| Risky sex | -0.35 | 0.46 | -0.76 | .45 |
| Sensation seeking*Social B-Do | 0.43 | 0.31 | 1.34 | .16 |
| Sensation seeking*Substance use | 0.34 | 0.75 | 0.53 | .60 |
| Sensation seeking*Risky drinking | -1.07 | 0.73 | -1.47 | .14 |
| Sensation seeking*Risky sex | 2.03 | 0.83 | 2.45 | .01 |
| Social B-Do*Substance use | 0.08 | 0.22 | 0.35 | .72 |
| Social B-Do*Risky drinking | -0.10 | 0.21 | -0.48 | .63 |
| Social B-Do*Risky sex | 0.21 | 0.25 | 0.85 | .40 |
| Sensation seeking* Social B-Do*Substance use | -0.34 | 0.37 | -0.85 | .40 |
| Sensation seeking* Social B-Do*Risky drinking | 0.44 | 0.36 | 1.24 | .21 |
| Sensation seeking* Social B-Do*Risky sex | -1.01 | 0.44 | -2.30 | .02 |
| Total Observations = 474 | | | | |
| **Random effects** | **Variance** | | ***S.D.*** | |
| Participant (intercept) | 0.046 | | 0.21 | |
| **Simple slopes** | **Estimate** | ***SE*** | ***t*** | ***p*** |
| **Aggressive and illegal behaviours** | | | | |
| Social B-Do (-1 SD) | -0.5 | 0.26 | 0.19 | .85 |
| Social B-Do (Mean) | 0.41 | 0.22 | 1.89 | .06 |
| Social B-Do (+1 SD) | 0.77 | 0.40 | 1.92 | .06 |
| **Substance use** | | | | |
| Social B-Do (-1 SD) | 0.06 | 0.31 | 0.20 | .84 |
| Social B-Do (Mean) | 0.14 | 0.20 | 0.70 | .48 |
| Social B-Do (+1 SD) | 0.22 | 0.24 | 0.90 | .37 |
| **Risky drinking** | | | | |
| Social B-Do (-1 SD) | -0.52 | 0.31 | -1.66 | .10 |
| Social B-Do (Mean) | 0.22 | 0.21 | 1.04 | .30 |
| Social B-Do (+1 SD) | 0.95 | 0.21 | 4.47 | <.001 |
| **Risky sex** | | | | |
| Social B-Do (-1 SD) | 0.93 | 0.33 | 2.82 | .01 |
| Social B-Do (Mean) | 0.44 | 0.23 | 1.89 | .06 |
| Social B-Do (+1 SD) | -0.14 | 0.38 | -0.12 | .91 |
| **Table S6: Estimates for Model 16.** This model included the main effect of age, gender, IQ and group, as well as an interaction between sensation seeking, perceived social benefit, and risk domain. | | | | |
